# High expression FUT1 and B3GALT5 is an independent predictor of postoperative recurrence and survival in hepatocellular carcinoma

**DOI:** 10.1038/s41598-017-11136-w

**Published:** 2017-09-07

**Authors:** Huan-Hsien Kuo, Ruey-Jen Lin, Jung-Tung Hung, Chung-Bao Hsieh, Tsai-Hsien Hung, Fei-Yun Lo, Ming-Yi Ho, Chau-Ting Yeh, Yen-Lin Huang, John Yu, Alice L. Yu

**Affiliations:** 1 Institute of Stem Cell & Translational Cancer Research, Chang Gung Memorial Hospital at Linkou and Chang Gung University, Taoyuan, Taiwan; 2 Institute of Biochemistry and Molecular Biology, National Yang-Ming University, Taipei, Taiwan; 3 Division of General Surgery, Department of Surgery, Tri-Service General Hospital, National Defense Medical Center, Taipei, Taiwan; 4 Liver Research Center, Department of Hepato-Gastroenterology, Chang Gung Memorial Hospital and College of Medicine, Chang Gung University, Taoyuan, Taiwan; 5 Department of Anatomic Pathology, Chang Gung Memorial Hospital, Taoyuan, Taiwan; 6 Department of Pediatrics, University of California in San Diego, San Diego, CA USA

## Abstract

Cancer may arise from dedifferentiation of mature cells or maturation-arrested stem cells. Previously we reported that definitive endoderm from which liver was derived, expressed Globo H, SSEA-3 and SSEA-4. In this study, we examined the expression of their biosynthetic enzymes, FUT1, FUT2, B3GALT5 and ST3GAL2, in 135 hepatocellular carcinoma (HCC) tissues by qRT-PCR. High expression of either FUT1 or B3GALT5 was significantly associated with advanced stages and poor outcome. Kaplan Meier survival analysis showed significantly shorter relapse-free survival (RFS) for those with high expression of either FUT1 or B3GALT5 (*P* = 0.024 and 0.001, respectively) and shorter overall survival (OS) for those with high expression of B3GALT5 (*P* = 0.017). Combination of FUT1 and B3GALT5 revealed that high expression of both genes had poorer RFS and OS than the others (*P* < 0.001). Moreover, multivariable Cox regression analysis identified the combination of B3GALT5 and FUT1 as an independent predictor for RFS (HR: 2.370, 95% CI: 1.505–3.731, *P* < 0.001) and OS (HR: 2.153, 95% CI: 1.188–3.902, *P* = 0.012) in HCC. In addition, the presence of Globo H, SSEA-3 and SSEA-4 in some HCC tissues and their absence in normal liver was established by immunohistochemistry staining and mass spectrometric analysis.

## Introduction

Liver cancer is the sixth most common cancer and the third leading cause of cancer-related deaths worldwide. The majority of primary liver cancers are hepatocellular carcinoma (HCC), which arises from hepatocytes. Hepatitis B virus (HBV) infection and aflatoxin B exposure are the two critical pathogenic factors leading to HCC in Eastern Asia and sub-Saharan Africa. In contrast, hepatitis C virus (HCV) infection is the major risk factor in Northern America, Europe and Japan^1–3^. Patients with advanced unresectable HCC had dismal outcomes with estimated survival rates of 17.5% at 1 year and 7.3% at 2 years^4^. Although patients with early-stage HCC are amenable to treatment by surgical resection, nonsurgical ablation procedures or liver transplantation, the overall survival remains poor because of high recurrence rate. Patients who received surgical resection had approximately 70% recurrence rate at 5 years^5^. Thus, identification of biomarkers for predicting relapse of patients with HCC is important. So far, vascular invasion, large tumor size and tumor multiplicity have been considered as poor prognostic factors for HCC recurrence after surgical treatments^6^. With the use of a variety of molecular profiling, including oligonucleotide microarray, quantitative reverse-transcription polymerase chain reaction (qRT-PCR), mass spectrometry, several biomarker candidates associated with HCC recurrence have been reported^7^. However, none of the candidate biomarkers have been put into clinical practice.

To date, no study has focused on the prognostic values of glycan structures or genes involved in their biosynthesis, even though aberrant glycosylation on glycoproteins or glycolipids is a common feature of cancer cells. These abnormal glycoconjugates contribute to tumorigenic processes such as proliferation and invasion of cancer cells, angiogenesis, immune modulation and metastasis^8, 9^. Many tumor-associated carbohydrate antigens have been identified such as polysialic acid, sialy-Lewis antigens, Thomsen-Friedenreich antigen, Thomsen-nouvelle antigen (Tn), saialy-Tn, GD2, GD3 and Globo H^10^. Globo H and stage-specific embryonic antigen-3 (SSEA-3), SSEA-4 are globoseries glycosphingolipids (GSLs). SSEA-3, also known as globopentaose (Gb5), is the precursor of SSEA-4 and Globo H. It is derived from the transfer of galactose to terminal GalNAc of Gb4 globoside by Beta-1,3-galactosyltransferase 5 (B3GALT5)^11^. Addition of a α2-3-linked sialic acid to SSEA-3 by ST3 beta-galactoside alpha-2,3-sialyltransferase 2 (ST3GAL2) gives rise to SSEA-4^12^, whereas addition of a α1-2-linked fucose to SSEA-3 by fucosyltransferase 1 (FUT1) or FUT2 generates Globo H^13^. SSEA-3 and SSEA-4 are well-known markers for human embryonic stem cells and can also be found on human teratocarcinoma cells^14, 15^. Several recent studies reported the expression of SSEA-3 in colorectal cancer^16^ and breast cancer stem cells^13^; expression of SSEA-4 in renal cell carcinoma^17^, epithelial ovarian carcinoma^18^, oral cancer^19^, glioblastoma multiforme^20^ and basaloid lung cancer^21^. Globo H is expressed in various epithelial cancers, including breast, colon, ovarian, gastric, pancreatic, lung and prostate cancers^22, 23^. On the other hand, few studies have examined the expression of SSEA-4 and Globo H in HCC. The presence of SSEA-4 was reported on both Hep-11 and Hep-12 cell lines derived from the primary and recurrent HCC tissues, respectively, of a single patient^24^. Globo H has been detected in the majority of 32 HCC tumor specimens by mass spectrometry^25^. Immunohistochemistry (IHC) analysis of a 16 HCC associated with HBV revealed expression of Globo H in 12 specimens^26^. These limited studies have shown the presence of SSEA-4 and Globo H in HCC tissues, but the expression of SSEA-3 has yet to be deciphered.

Previously, we have demonstrated that the GSL profiles of human embryonic stem cell changed dramatically during differentiation into different lineages. Definitive endoderm, but not neuroectoderm, expressed several globosides and lactosides, which was consistent with higher expression levels of FUT1, FUT2 and B3GALT5 in endodermal lineage than those in neural progenitor cells^27^. The definitive endoderm is one of the primary germ layers that participates in the complex morphogenesis of the gut tube, which is the primitive precursor of liver, thymus, lung, pancreas, prostate, GI tract and thyroid^28^. Since liver cells are derived from endoderm, these glycosyltransferases may also play important roles in HCC. The reported detection of Globo H and SSEA-4 in HCC suggests the possibility of increased expression of their biosynthetic enzymes FUT1/FUT2 and ST3GAL2, respectively, and perhaps, B3GALT5, which is involved in the generation of their precursor, SSEA-3. However, the clinical relevance of these globoside biosynthetic enzymes has not been addressed.

In the present study, we examined the mRNA levels of glycosyltransferases, including FUT1, FUT2, B3GALT5 and ST3GAL2, responsible for the synthesis of Globo H, SSEA-3 and SSEA-4 in 135 HCC specimens by qRT-PCR and correlated with clinical-pathological parameters and outcomes of patients. Our data demonstrated for the first time that patients with higher expression of the FUT1 and B3GALT5, had shorter relapse-free survival (RFS). In addition, we unveiled the expression of SSEA-3 and validated the presence of Globo H, SSEA-3 and SSEA-4 in HCC tissues, but not in normal liver, by IHC staining and mass spectrometry analysis.

## Results

### Expression of FUT1, FUT2, B3GALT5 and ST3GAL2 in HCC tissues

**Figure 1:**
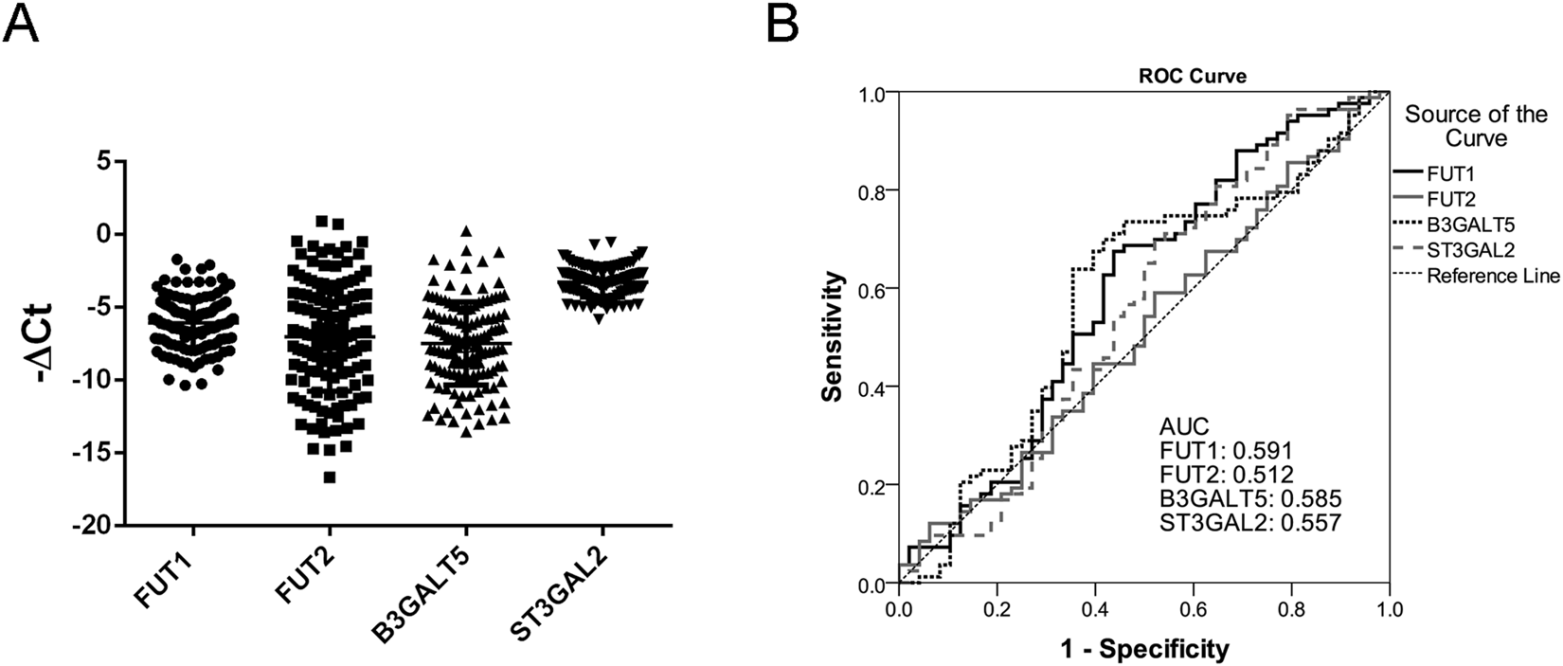
Expression levels of FUT1, FUT2, B3GALT5 and ST3GAL2 in HCC tissues and their ROC analyses in relation to disease recurrence. (**A**) The RNA expression levels of FUT1, FUT2, B3GLAT5 and ST3GAL2 in HCC tissues were determined by qRT-PCR. The CT value of each specimen was normalized to the average of control and presented as minus delta threshold cycle (−ΔCt). (**B**) The values of −ΔCt of FUT1, FUT2, B3GLAT5 and ST3GAL2 were used to plot the ROC curves to predict the relapse of patients with HCC. AUC: area under curve.

Since cancer cells may arise from maturation arrest of immature stem cells or from dedifferentiation of mature cells^29^, it is likely that Globo H and its related biosynthetic enzymes expressed on endodermal cells might also be expressed on liver cancer cells. We examined the mRNA expression levels of the FUT1, FUT2, B3GALT5 and ST3GAL2 in tumor specimens of 135 HCC patients by qRT-PCR. Their clinical characteristics and demographic information were summarized in Table 1. The mean age was 57.7 ± 13.1 (range: 19–87) with 82.2% being male. All except 5 of 135 patients had chronic hepatitis infection with a majority (70.4%) infected by HBV. Six patients were infected by both HBV and HCV and remaining were infected by HCV. Liver cirrhosis was found in 59 cases (43.7%). Tumor sizes ranged from 0.8 to 16 cm with a mean size of 5.4 ± 3.9 cm, but less than 5 cm in 65.2%. Furthermore, 74 patients (54.8%) had histological grade 1–2 tumors and 66 patients (48.9%) showed evidence of vascular invasion in their tumors. According to the tumor-node-metastasis (TNM) system, 102 patients (75.6%) presented with TNM I + II stages. Thirteen patients (9.6%) had tumor metastasis at diagnosis. The median follow-up time was 58.6 months (range, 1.4 to 126.24 months). At the time of this report, 47 patients (34.8%) died and the 1 y, 3 y and 5 y overall survival rate was 87.2%, 73.7% and 66.4%, respectively. All deaths were related to HCC except three patients who died of other diseases. The results of qRT-PCR analyses of the 4 globoside biosynthetic genes in these samples were shown as −ΔCT, after subtracting the average of 2 reference genes, GAPDH and GUSB (Fig. 1A). The mean expression level was −6.05 ± 1.71 (−10.34 to −1.70) for FUT1, −7.02 ± 3.74 (−16.67 to 0.92) for FUT2, −7.48 ± 2.85 (−13.53 to 0.25) for B3GALT5 and −3.28 ± 1.02 (−5.83 to −0.53) for ST3GAL2. The expression levels of these 4 genes varied greatly among patients (ANOVA, *P* < 0.001) and ST3GAL2 expression was significantly higher than the other three genes.

**Table 1:** Clinical and pathological characteristics of 135 HCC patients.

Characteristics	N (%)
Age (Mean ± SD and range) (years)	57.7 ± 13.1 (19–87)
Gender	
Male	111 (82.2%)
Female	24 (17.8%)
Drinking history	
Yes	43 (31.9%)
No	92 (68.1%)
Virus infection	
None	5 (3.7%)
HBV	95 (70.4%)
HCV	29 (21.5%)
HBV + HCV	6 (4.4%)
Tumor size (Mean ± SD and range) (cm)	5.4 ± 3.9 (0.8–16)
≦5	88 (65.2%)
>5	47 (34.8%)
Serum AFP (ng/ml)^a^	
≦200	87 (65.4%)
>200	46 (34.6%)
Edmondson Grade	
1 + 2	74 (54.8%)
3 + 4	61 (45.2%)
TNM stage^b^	
I + II	102 (75.6%)
III + IV	33 (24.4%)
Vascular invasion	
Absent	69 (51.1%)
Present	66 (48.9%)
Tumor number	
Solitary	84 (62.2%)
Multiple	50 (37%)
Diffuse infiltrative	1 (0.7%)
Cirrhosis	
No	76 (56.3%)
Yes	59 (43.7%)
Metastasis	
No	122 (90.4%)
Yes	13 (9.6%)
Relapse^c^	
No	48 (36.6%)
Yes	83 (63.4%)
RFS duration median (range)	27.3 (0.7–114.7 months)
Outcomes	
Alive	88 (65.2%)
Dead	47 (34.8%)
OS duration median (range)	58.6 (1.4–126.6 months)

*AFP* alpha-fetoprotein, *HBV* hepatitis B virus, *HCV* hepatitis C virus, *TNM* tumor-node-metastasis, *RFS* relapse-free survival, *OS* overall survival. ^a^Data not available in 2 patients, ^b^According to the American Joint Committee on Cancer (AJCC) TNM staging system 7th edition (2010), ^c^Tumor persistence in 4 patients.

### Correlation of clinicopathological parameters with the expression levels of FUT1, FUT2, B3GALT5 and ST3GAL2

To explore the clinical relevance of the expression levels of these 4 globoside glycosyltransferases, we used the area under curve (AUC) of receiver operating characteristic (ROC) curve to evaluate their predictive values for disease recurrence. Among 135 patients, 4 patients who had persistent disease were excluded from relapse analyses, and 83 of 131 patients (63.4%) had disease recurrence. Since the date of relapse was not available in 2 patients and one patient in remission died of unrelated cause, the ensuing RFS analyses were conducted in 128 patients. As shown in Fig. 1B, the AUCs for FUT1, FUT2, B3GALT5 and ST3GAL2 were 0.591, 0.512, 0.585 and 0.557, respectively. Based on these findings, we further analyzed the prognostic significance of their expression levels in HCC, using Youden index to determine the optimal cutoff values defining high and low expression groups. The best cutoff value for predicting recurrence of HCC was −6.433 for FUT1, −7.633 for FUT2, −7.724 for B3GALT5 and −3.652 for ST3GAL2. The association of expression of each of the four genes with clinical pathological parameters in 135 patients with HCC was analyzed (Table 2). The expression levels of FUT1 were significantly correlated with TNM stage (*P* = 0.002), tumor number (*P* = 0.001), vascular invasion (*P* = 0.039), metastasis (*P* = 0.011) and relapse (*P* = 0.008). Patients with High expression of FUT1 were at higher risk than those with low expression of FUT1 for having TNM III + IV stages (OR: 4.16, 95% CI: 1.58–10.93), multiple tumors (OR: 3.90, 95% CI: 1.76–8.63) presence of vascular invasion (OR: 2.08, 95% CI: 1.03–4.20), metastasis (OR: 9.52, 95% CI: 1.20–75.59) and relapse (OR: 2.66, 95% CI: 1.28–5.54). The expression of B3GALT5 showed association with TNM stage (*P* < 0.001), vascular invasion (*P* = 0.020), metastasis (*P* = 0.002), relapse (*P* = 0.002) and survival (*P* = 0.006). In addition, the odds ratio for TNM III + IV stage, vascular invasion, metastasis, relapse and survival was 6.17 (95% CI: 2.35–16.26), 2.26 (95% CI: 1.13–4.51), 13.24 (95% CI: 1.67–105), 3.22 (95% CI: 1.53–6.77) and 2.81 (95% CI: 1.33–5.91), respectively. The ST3GAL2 showed a significant association with number of tumor nodules (*P* = 0.003), Edmonson-Steiner grade (*P* = 0.008) and relapse (*P* = 0.042). When compared to low expression of ST3GAL2, patients with high expression of ST3GAL2 were at higher risk for multiple tumors (OR: 3.22, 95% CI: 1.46–7.13), moderately and poorly differentiated tumor (OR: 2.66, 95% CI: 1.28–5.53) and relapse (OR: 2.13, 95% CI: 1.02–4.45). On the other hand, the FUT1, B3GALT5, ST3GAL2 expression levels were not significantly associated with age, virus infection, liver cirrhosis or AFP. As to FUT2, there was no significant association with any clinicopathological features (Supplementary Table 1). These findings revealed that high expression of FUT1 or B3GALT5 was significantly associated with advanced tumor stage and metastatic phenotype of HCC.

**Table 2:** Association of FUT1, B3GALT5, and ST3GAL2 expression with clinical-pathological parameters in 135 patients with HCC.

Variable	N	FUT1				B3GALT5				ST3GAL2			
		Low	High	*P* value^a^	OR (95% CI)	Low	High	*P* value^a^	OR (95% CI)	Low	High	*P* value^a^	OR (95% CI)
Age													
<55	56	23	33	0.948	1.0	25	31	0.493	1.0	23	33	0.608	1.0
≧55	79	32	47		1.02 (0.51–2.05)	40	39		0.79 (0.40–1.56)	29	50		1.20 (0.60–2.43)
Gender													
Female	24	9	15	0.722	1.0	7	17	**0.040**	1.0	6	18	0.133	1.0
Male	111	46	65		0.84 (0.34–2.10)	58	53		0.38 (0.15–0.98)	46	65		0.47 (0.17–1.28)
Virus infection													
None	5	2	3	0.986	1.0	1	4	0.311	1.0	1	4	0.323	1.0
HBV	95	39	56		0.96 (0.15–6.00)	50	45		0.23 (0.02–2.09)	41	54		0.33 (0.03–3.06)
HCV	29	12	17		0.94 (0.14–6.54)	11	18		0.41 (0.04–4.15)	9	20		0.56 (0.05–5.70)
HBV + HCV	6	2	4		1.33 (0.11–15.70)	3	3		0.25 (0.17–3.77)	1	5		1.25 (0.06–26.87)
Liver cirrhosis													
No	76	35	41	0.154	1.0	38	38	0.625	1.0	32	44	0.331	1.0
Yes	59	20	39		1.66 (0.82–3.36)	27	32		1.19 (0.60–2.34)	20	39		1.42 (0.70–2.87)
TNM stage													
I + II	102	49	53	**0.002**	1.0	59	43	<**0.001**	1.0	42	60	0.265	1.0
III + IV	33	6	27		4.16 (1.58–10.93)	6	27		6.17 (2.35–16.26)	10	23		1.61 (0.70–3.73)
Tumor size (cm)													
≦5	88	39	49	0.247	1.0	47	41	0.094	1.0	35	53	0.682	1.0
>5	47	16	31		1.54 (0.73–3.21)	18	29		1.85 (0.90–3.80)	17	30		1.17 (0.56–2.42)
AFP (ng/mL)^b^													
≧200	87	34	53	0.803	1.0	43	44	0.679	1.0	35	52	0.539	1.0
>200	46	19	27		0.91 (0.44–1.88)	21	25		1.16 (0.57–2.38)	16	30		1.26 (0.60–2.65)
Tumor number^c^													
Solitary	84	44	40	**0.001**	1.0	43	41	0.421	1.0	40	44	**0.003**	1.0
Multiple	50	11	39		3.90 (1.76–8.63)	22	28		1.34 (0.66–2.70)	11	39		3.22 (1.46–7.13)
Grade													
1–2	74	32	42	0.515	1.0	33	41	0.363	1.0	36	38	**0.008**	1.0
3–4	61	23	38		1.259 (0.63–2.51)	32	29		0.73 (0.37–1.44)	16	45		2.66 (1.28–5.53)
Vascular invasion													
Absent	69	34	35	**0.039**	1.0	40	29	**0.020**	1.0	26	43	0.838	1.0
Present	66	21	45		2.08 (1.03–4.20)	25	41		2.26 (1.14–4.51)	26	40		0.93 (0.47–1.86)
Metastasis													
No	122	54	68	**0.011**	1.0	64	58	**0.002**	1.0	50	72	0.071	1.0
Yes	13	1	12		9.52 (1.20–75.59)	1	12		13.24 (1.67–105).	2	11		3.82 (0.81–17.98)
Relapse^d^													
No	48	27	21	**0.008**	1.0	31	17	**0.002**	1.0	23	25	**0.042**	1.0
Yes	83	27	56		2.66 (1.28–5.54)	30	53		3.22 (1.53–6.77)	25	58		2.13 (1.02–4.45)
Survival													
Alive	88	41	47	0.058	1.0	50	38	**0.006**	1.0	33	55	0.74	1.0
Death	47	14	33		2.05 (0.96–4.36)	15	32		2.81 (1.33–5.91)	19	28		0.88 (0.43–1.83)

*HBV* hepatitis B virus, *HCV* hepatitis C virus, *TNM* tumor-node-metastasis, *AFP* alpha-fetoprotein, *OR* Odds Ratio. ^a^Pearson Chi-square test. ^b^Data not available in 2 patients. ^c^One patient with diffuse infiltrating tumor was excluded, ^d^4 patients with persistent tumor were excluded. Statistically significant values are displayed in boldface.

### High expression levels of FUT1 and B3GALT5 correlate with poor clinical outcome

**Figure 2:**
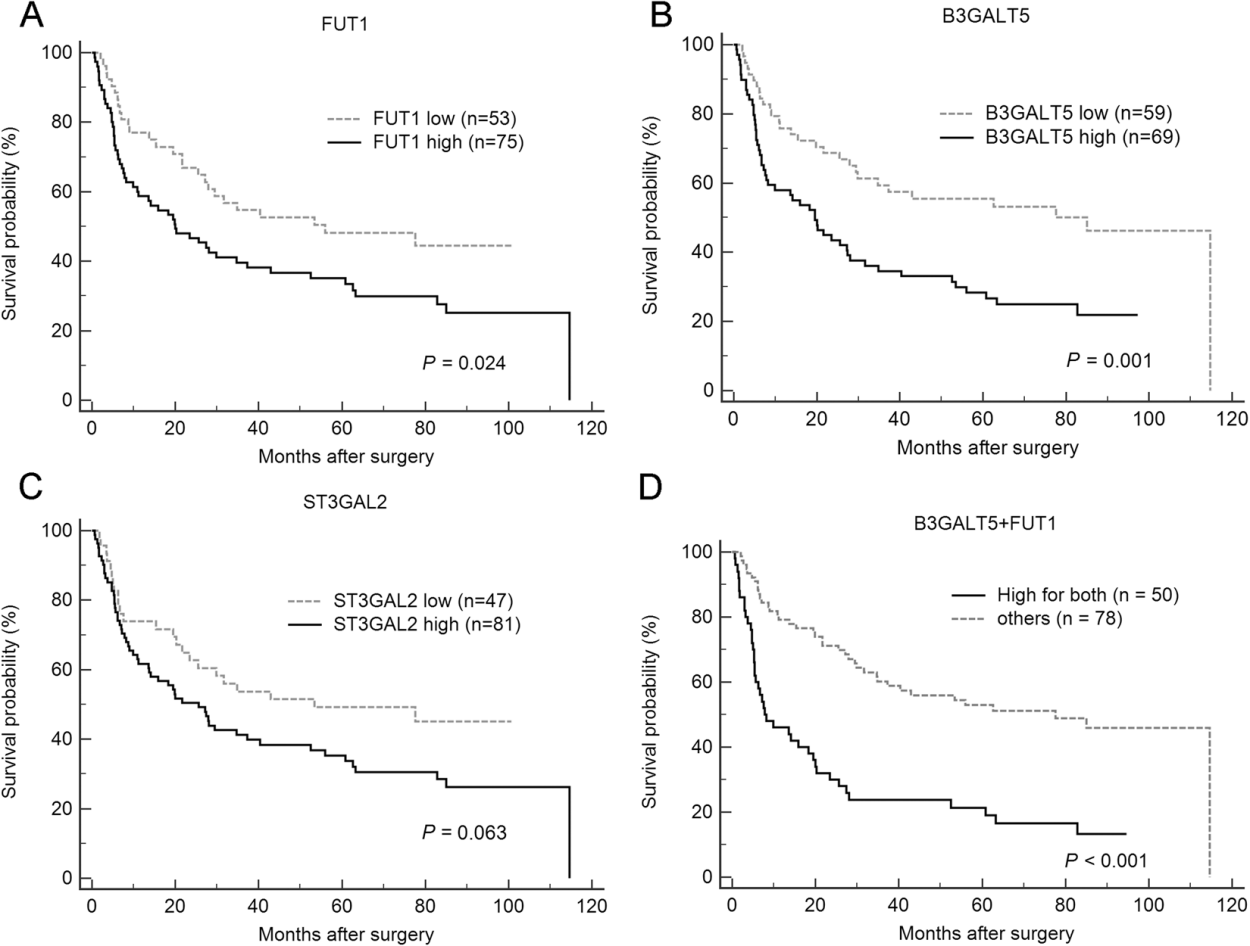
Kaplan-Meier plots of RFS for patients with HCC in relation to FUT1, B3GALT5, and ST3GAL2 expression level. Patients with high level of FUT1 (**A**), B3GALT5 (**B**), ST3GAL2 (**C**), and FU1 + B3GALT5 combined (**D**) had poorer RFS. Seven of 135 patients were excluded from relapse analyses: 4 patients who had persistent disease, one patient who died of unrelated cause while in remission, and 2 patients for whom the date of relapse was not available.

**Figure 3:**
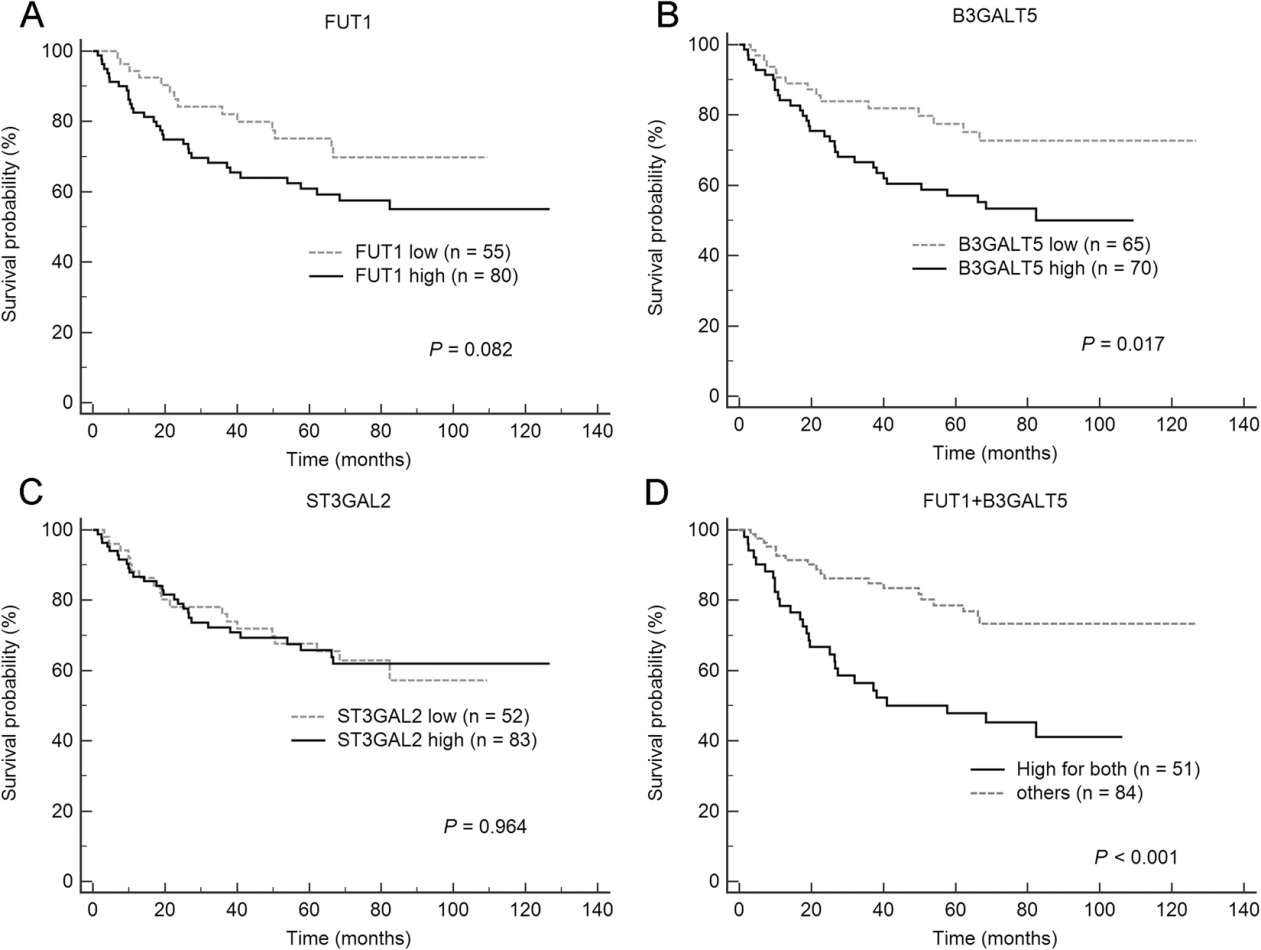
Kaplan-Meier plots of overall survival for patients with HCC in relation to the expression levels of FUT1 (**A**), B3GALT5 (**B**), ST3GAL2 (**C**) and FUT1 + B3GALT5 combined (**D**).

We further investigated whether high expression of FUT1, B3GALT5 and ST3GAL2 was a significant predictor of recurrence of HCC. Kaplan-Meier analysis revealed that RFS in patients with high expression of FUT1 (median: 20.0, 95% CI: 10.86–37.29 months), and B3GALT5 (median: 19.5, 95% CI: 7.8–28.07 months) was significantly shorter than those with low expression of FUT1 (median: 56.0, 95% CI: 27.20–77.64 months; *P* = 0.024) and B3GALT5 (median: 85.1, 95% CI: 29.80–114.68 months; *P* = 0.001) (Fig. 2). Patients with high expression of ST3GAL2 tend to have shorter RFS, albeit the difference did not reach statistical significance (*P* = 0.063). Kaplan-Meier analyses of overall survival (OS) showed that patients with high expression of B3GALT5 had significantly poorer OS than those with low expression (*P* = 0.017), whereas the levels of FUT1 and ST3GAL2 did not significantly correlate with OS (Fig. 3).

To further evaluate the joint effects of FUT1 and B3GALT5, patients were stratified into three groups: high for both, low for both and the others, for Kaplan-Meier survival analysis. The RFS and OS were not statistically different between the low for both group and others (*P* = 0.303 and 0.195, respectively) (supplementary Fig. 1s). Thus, these two groups were combined into one for comparison with the high for both group. As shown in Fig. 2D, patients with high expression of FUT1 and B3GALT5 combined had significantly lower rate of RFS than the remaining group (*P* < 0.001), at 1 year (46.0% vs. 79.2%), 3 years (23.7% vs. 60.2%), and 5 years (21.3% vs. 52.9%). In addition, these patients also had significantly shorter OS than the remaining group (*P* < 0.001) at 1 year (78.4% vs. 92.7%), 3 years (56.4% vs. 84.8%) and 5 years (47.8 vs. 78.6%) (Fig. 3D).

### High expression of FUT1 and B3GALT5 combined is an independent prognostic factor for HCC relapse and overall survival

To evaluate the potential value of the expression levels of FUT1, B3GALT5 and ST3GAL2 for predicting HCC recurrence and OS, univariate Cox proportional hazard regression analyses were conducted. The results indicated that RFS correlated with the patient older than 55-year (HR: 1.960, 95% CI: 1.233–3.126, *P* = 0.005), tumor size greater than 5 cm (HR: 3.113, 95% CI: 1.975–4.906, *P* < 0.001), presence of metastasis (HR: 3.632, 95% CI: 1.891–6.973, *P* < 0.001), and presence of vascular invasion (HR: 3.317, 95% CI: 2.098–5.244, *P* < 0.001). In addition, RFS duration of HCC patients was significantly associated with high expression of FUT1 (HR: 1.698, 95% CI: 1.067–2.701, *P* = 0.025) and B3GALT5 (HR: 2.082, 95% CI: 1.313–3.302, *P* = 0.002). Similarly, the above-mentioned factors except FUT1 also significantly correlated with OS (Table 3). Next, to identify the independent variables associated with poor RFS and OS, we selected those important covariates which showed statistical significance in the univariate analysis for multivariable Cox regression analysis in a stepwise manner. As shown in Table 3 in addition to age ≥55, tumor ≥5 cm and vascular invasion, the expression levels FUT1 and B3GALT5 combined were independent risk factors for both RFS (HR: 2.370, 95% CI: 1.505–3.731, *P* < 0.001) and OS (HR: 2.153, 95% CI: 1.188–3.902, *P* = 0.012) in patients with HCC. These findings identified the combined expression of FUT1 and B3GALT5 as an important and independent predictor for RFS and OS in HCC patients.

**Table 3:** Univariate and multivariable Cox proportional regression analysis of factors associated with HCC recurrence and survival.

variables	RFS						OS					
	Univariate analysis			Multivariable analysis			Univariate analysis			Multivariable analysis		
	HR	95% CI	*P-value*	HR	95% CI	*P-value*	HR	95% CI	*P-value*	HR	95% CI	*P-value*
Age: ≥55 vs. <55	1.960	1.223–3.126	**0.005**	1.875	1.173–2.999	**0.009**	2.177	1.150–4.120	**0.017**	2.096	1.095–4.011	**0.026**
Gender: male vs. female	0.709	0.410–1.228	0.220				0.592	0.308–1.140	0.119			
Hepatitis virus infection: yes vs. no	0.801	0.293–2.190	0.665				1.712	0.239–12.293	0.595			
Cirrhosis: present vs. absent	1.237	0.797–1.920	0.344				0.815	0.454–1.465	0.496			
Tumor size (cm): ≥5 vs. <5	3.113	1.975–4.906	<**0.001**	2.042	1.248–3.344	**0.005**	4.283	2.384–7.694	<**0.001**	2.728	1.452–5.126	**0.002**
AFP (ng/ml): ≥200 vs. <200	1.128	0.707–1.800	0.612				1.291	0.711–2.344	0.403			
Tumor number: mutiple vs. solitary	1.328	0.845–2.087	0.219				1.623	0.910–2.897	0.103			
Grade: III + IV vs I + II	0.888	0.571–1.382	0.600				0.651	0.357–1.188	0.164			
Vascular invasion: absent vs. present	3.317	2.098–5.244	<**0.001**	2.173	1.323–3.570	**0.002**	5.305	2.638–10.665	<**0.001**	2.792	1.305–5.975	**0.008**
Metastasis: yes vs. no	3.632	1.891–6.973	<**0.001**			NS	4.207	2.084–8.493	<**0.001**			NS
FUT1: high vs. low	1.698	1.067–2.701	**0.025**			NS	1.731	0.929–3.224	0.086			
B3GALT5: high vs. low	2.082	1.313–3.302	**0.002**			NS	2.081	1.130–3.831	**0.019**			NS
ST3GAL2: high vs low	1.568	0.971–2.533	0.066				0.987	0.552–1.763	0.965			
FUT1 + B3GALT5 combined: high for both vs others	2.927	1.877–4.564	<**0.001**	2.370	1.505–3.731	<**0.001**	2.908	1.627–5.196	<**0.001**	2.153	1.188–3.902	**0.012**

*RFS* relapse-free survival*, OS* overall survival*, HR* hazard ratio, *CI* confidence interval, *NS* not significant; Multivariable modeling using stepwise variable selection. Statistically significant values are displayed in boldface.

### Determination of the expression of Globo H, SSEA-3 and SSEA-4 in HCC and normal liver tissues

**Figure 4:**
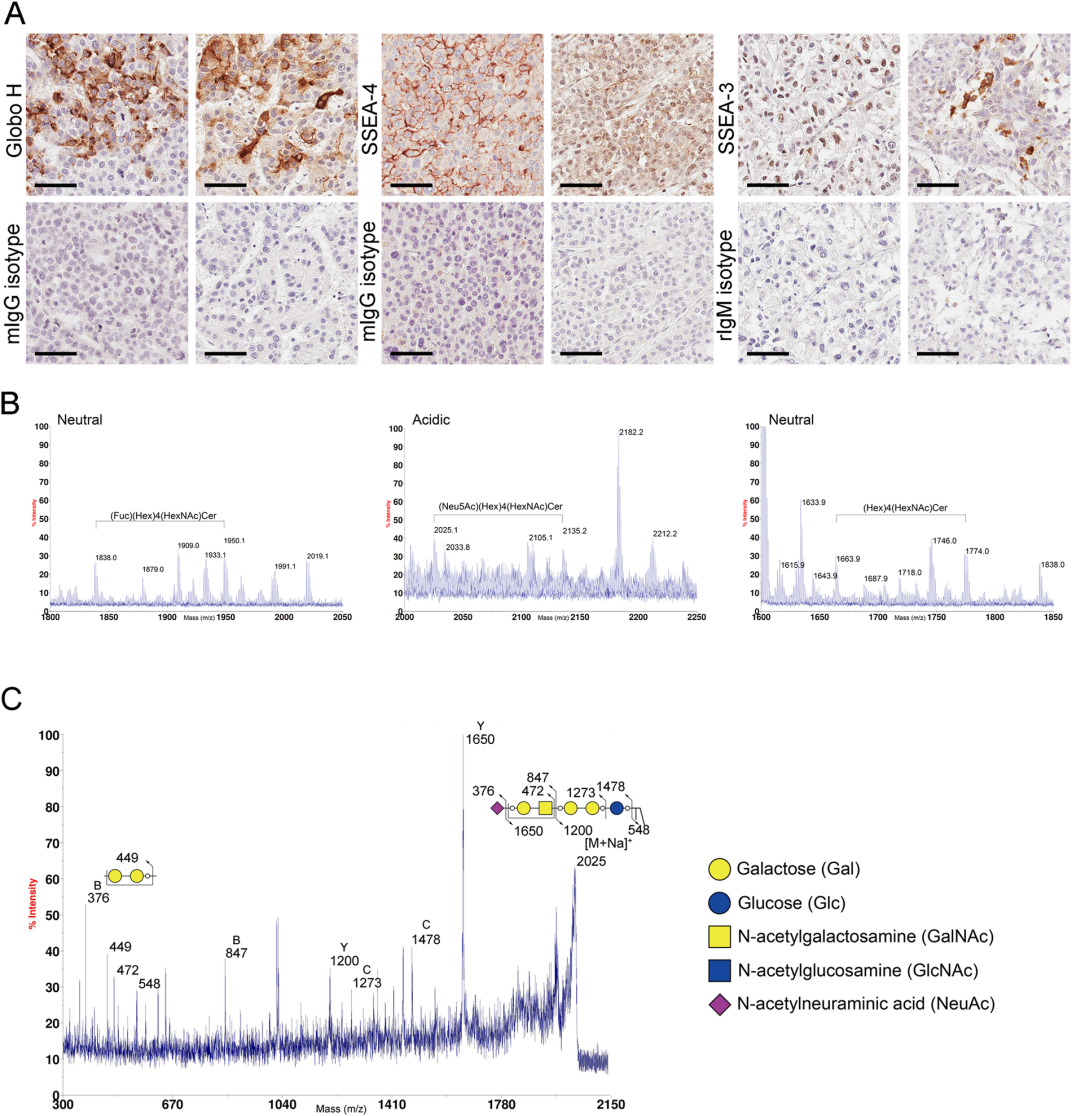
Expression of Globo H, SSEA-4 and SSEA-3 and in HCC. (**A**) Expression of Globo H, SSEA-4 and SSEA-3 in formalin-fixed, paraffin-embedded HCC tissue sections was determined by IHC staining with mAbs VK9, MC-813-70 and MC-631, respectively. Two representative positive stainings for each of the 3 globosides and their isotype controls were shown. Scale bars indicate 60 μm (original magnification 40X) (**B**) MALDI-TOF MS spectra of permethylated GSLs derived from an HCC tumor tissue which showed positive IHC stainings for Globo H, SSEA-4, and SSEA-3. Signals of Fuc_1_Hex_4_HexNAc_1_Cer (Globo H, *m/z* = 1,838/2,050) and Hex_4_HexNAc_1_Cer (SSEA-3, *m/z* = 1,664/1,774) were found in the neutral fraction. Neu5AC_1_Hex_4_HexNAcCer (SSEA-4, *m/s* = 2,025/2,135) was detected in the acidic fraction. These GSLs carried both C16:0 and C24:0/C24:1 ceramides (at 112/110 mass units higher than those with C16:0) (**C**) MALDI CID MS/MS spectra of the precursor at *m/z* 2,025 (SSEA-4). Assignments of fragment ions were shown on the illustrations. Only the O atoms at preferred cleavage sites were drawn out to distinguish between **B** and **C** ions.

Only a few studies reported the presence of SSEA-4 and Globo H in HCC tissues^25, 26, 30^ and the expression of SSEA-3 has yet to be deciphered. To examine the expression of SSEA-4, SSEA-3 and Globo H on HCC tissues, we obtained paraffin-embedded HCC tissue sections and performed IHC staining with monoclonal antibodies against the SSEA-4, SSEA-3 and Glob H, respectively. Positive IHC stainings from 2 representative HCC specimens for each glycan antigen were shown in Fig. 4A. In general, IHC staining patterns of all three globosides were heterogeneous. In most cases, Globo H positive cells were observed in clusters within the tumor but occasionally dispersed Globo H positive cells were also noted. The majority of Globo H positive cells displayed membranous and/or uneven cytoplasmic staining, while nuclear staining was rarely seen. SSEA-4 staining usually appeared in patches of uniformly staining cells with predominant membranous pattern and in some cases with nuclear staining. SSEA-3 positive cells were usually dispersed throughout the tumor, with discrete and granular staining of the whole cell in some cases and nuclear staining only in others. However, in light of the reported cross-reactivities of anti-glycan antibodies observed by glycan array assay^31^, we confirmed the presence of these glycans by mass spectrometric analysis of GSLs extracted from the tumor tissues that stained positively for Globo H, SSEA-4 or SSEA-3 on IHC. Purified GSLs were analyzed by MALDI-TOF mass spectrometry. On the basis of the *m/z* values of major molecular ion signals and the usual ranges of common permutations of sphingosine and fatty acyl along with the characteristics of GSLs, the signals of FucHex_4_HexNAc_1_Cer (*m/z* = 1,838/1,950) and Hex_4_HexNAc_1_Cer (*m/z* = 1,664/1,774) that represented Globo H and SSEA-3 were found in the neutral fraction. In the acidic fraction, the signals with Neu5Ac_1_Hex_4_HexNAc_1_Cer (*m/z* = 2,025/2,135) which corresponded to SSEA-4 were detected (Fig. 4B). The molecular ions at *m/z* 2,025 were further confirmed by additional MS/MS analysis. The major afforded fragment ions were annotated, including the ion at *m/z* 548 which revealed the ceramide moiety, B ions at *m/z* 376 and 847 corresponding to terminal Neu5Ac and Neu5ACHexHexNAc, Y ion at 1,650 deriving from loss of Neu5Ac and C ion at *m/z* 1,478 produced through glycosidic cleavage at glucose extending from ceramide. Ions at *m/s* 449 and 472 corresponding to internal Hex_2_ and HexHexNAc units were also detected. These MS/MS data identified the molecular ion signals at *m/z* 2,025 as SSEA-4 (Fig. 4C). Recently, *Zhu et al*. demonstrated that Globo H was detected in HCC tissues by mass spectrometry. We also identified Globo H in HCC by using leech ceramide glycanase to release glycans from GSLs, and then analyzed by LC MS/MS (Data not shown).

**Figure 5:**
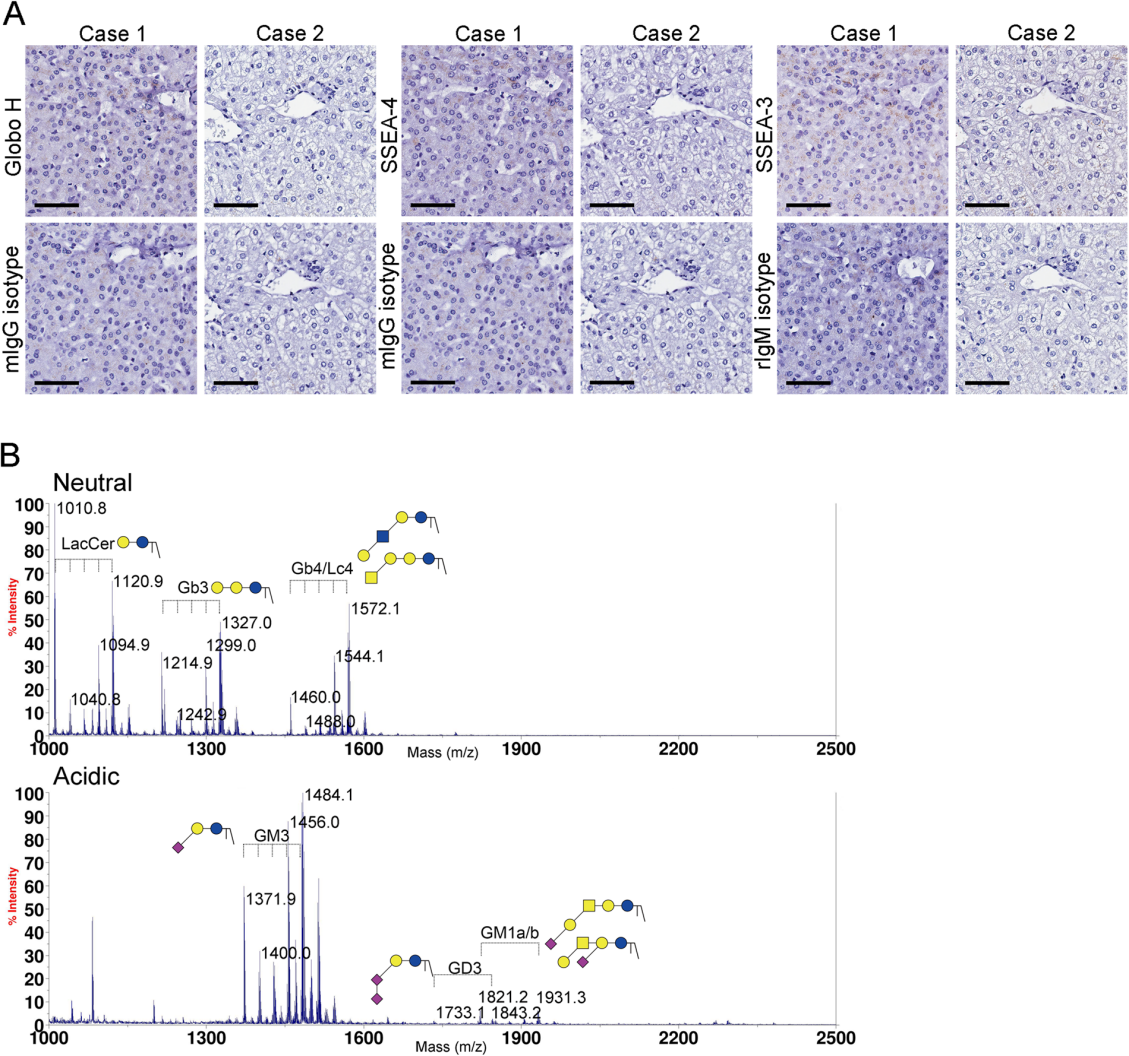
Lack of Globo H, SSEA-4 and SSEA-3 expression in normal liver tissue as determined by IHC and MALDI-MS analyses of permethylated GSLs. (**A**) Formalin-fixed, paraffin-embedded normal liver tissue sections from two cases were stained with mAbs VK9, MC-813-70 and MC-631 for the detection of Globo H, SSEA-4, and SSEA-3, respectively. Scale bars indicate 60 μm (original magnification 40X) (**B**) Total GSLs extracted from case 1 were separated into neutral and acidic fractions, permethylated and analyzed by MALDI-TOF mass spectrometry. The major GSLs in the neutral fraction were LacCer, Gb3, Gb4/nLc4, and GM3, GD3 and GM1a/b were found in the acidic fraction. GSLs with the same glycan moiety but with different fatty acyl contents were bracketed.

Next, we performed IHC to examine the expression of SSEA-3, -4 and Globo H in normal liver tissues obtained from two trauma patients. None of these three globosides were detected by IHC staining of normal liver, although weak non-specific granular signals were observed for SSEA-3 staining (Fig. 5A). This was further confirmed by MALDI-TOF MS analyses of the GSLs extracted from the same tissues, showing that the majority of GSLs in the neutral fraction were lactosylceramide (LacCer), globotriaosylceramide (Gb3) and globoside (Gb4)/ neolactotetraosylceramide (nLc4) (Fig. 5B). No signal for Globo H or SSEA-3 was detected. In the acidic fraction, the most abundant GSLs was GM3, and no SSEA-4 was detected. Taken together, Globo H, SSEA-3 and SSEA-4 are not expressed in normal liver tissues.

## Discussion

In this study, we provide the first evidence that expression level of FUT1, B3GALT5 and ST3GAL2, the enzymes catalyzing the synthesis of three globosides, Globo H, SSEA-3 and SSEA-4, was associated with adverse clinical features. Greater expression of either FUT1 or B3GALT5 in HCC tissue was associated with poor prognosis. More importantly, high expression of these two genes combined emerged as an independent predictor for RFS and OS in HCC.

Overexpression of FUT1 had been reported in colon cancer^32^ and shown to confer resistance to serum starvation-induced cell death^33^. Since serum starvation elicits autophagy, the protection conferred by FUT1 may be explained by our recent findings that FUT1 impedes perinuclear localization of lysosomes via fucosylation of LAMP1 and LAMP2, thereby preventing autophagic death^34^. In addition, our findings in this study are consistent with several reports by others. Zhang *et al*. found that knockdown of FUT1 by siRNA in epidermoid carcinoma cell line A431 inhibited tumor growth in mice^35^. Goupille *et al*. observed increasing tumorigenicity *in vivo* of rat colon carcinoma cells transfected with FUT1^36^. Furthermore, Amin *et al*. demonstrated that FUT1 played an important role in angiogenesis *in vitro* and *in vivo* with the use of FUT1 null endothelial cells and FUT1 knockout mice^37^. Also, data mining of breast cancer by Milde-Langosch *et al*. showed that high expression of FUT1 was a poor prognostic factor for breast cancer and associated with distant metastasis^38^. These studies were in line with our findings that high expression of FUT1 in HCC was associated with vascular invasion and multiple tumor nodules. On the other hand, Mathieu *et al*. reported that introducing FUT1 into HepG2 cells inhibited angiogenesis and tumor growth^39^. However, the HepG2 cell is a hepatoblastoma^40^, which is an embryonal malignancy of hepatocellular origin and has different clinical features distinctive from HCC. Such differences may account for the differential roles of FUT1 in hepatoblastoma HepG2 cell and HCC.

Besides FUT1, we also showed high expression of B3GALT5 in HCC tissues to be associated with advanced TNM stage, metastasis, vascular invasion and tumor recurrence. Among the 6 members of beta 1–3 galactosyltransferase family, B3GALT5 has broadest range of acceptors. In addition to transferring a galactose to the terminal GalNAc of GB4 to generate SSEA-3, B3GALT5 is also responsible for the synthesis of type 1 chain (Galβ1-3GlcNAc), which is the precursor of Lewis A and sialyl Lewis A (SLe^a^), the latter is also known as CA19-9 antigen. Along this line, the expression of B3GALT5 was shown to be correlated with CA19-9 in pancreatic cell lines and cancer tissues^41, 42^. SLe^a^ is a ligand for selectins and mediates adhesion of cancer cell to endothelial cells and promotes tumor progression^43^. Similarly Chung *et al*.^44^ reported that overexpression of HBV X protein in Chang cell enhanced SLe^a^ expression and mediated cell adhesion to endothelial cell and metastasis through induction of B3GALT5. Thus, it is possible that high expression of B3GALT5 might promote tumor metastasis through SLe^a^ and other glycans in HCC tissue.

Moreover, we found that higher expression of ST3GALT2 was associated with the number of HCC tumor nodules and high histological grades. The molecular mechanisms by which ST3GAL2 directly or indirectly contributes to tumor progression are not clear. SSEA-4 is a well-known product generated by ST3GAL2. Although the biological function of SSEA-4 is still unclear, it has been identified as a marker for human embryonic stem cells as well as cancer stem cells in breast cancer^45^. In addition, Yang *et al*. reported that knockdown of ST3GAL2 significantly reduced the phosphorylation of ERK1/2 and EGFR in the osteoblasts^46^. Since EGFR pathway is known to contribute to the inflammation in the liver and hepatocarcinogenesis^47^, ST3GAL2 might promote tumorigenesis through EGFR pathway. Saito *et al*. reported higher expression of ST3GAL2 in renal cell carcinoma tissues than in non-tumor kidney, suggesting that ST3GAL2 might be involved in the malignant processes^12^. In addition, Aloia *et al*. identified a subpopulation of breast cancer cells carrying high levels of SSEA-4 to be resistant to chemotherapy drugs and showed ST3GAL2 to be a marker of poor outcome in breast cancer and ovarian cancer patients undergoing chemotherapy^48^. These reports are in line with our finding that high expression of ST3GAL2 is associated with HCC progression.

In this study, we provided the first evidence for SSEA-3 expression in HCC by immunohistochemistry and mass spectrometry. We also confirmed the presence of both SSEA-4 and Globo H in HCC tumor cells by IHC staining and mass spectrometric analysis of GSLs from HCC tumor tissues. Furthermore, none of these three globosides were found in normal liver tissues by either IHC or mass spectrometry. There are only a few studies addressing the biological functions of Globo H, SSEA3 and SSEA-4. Sorting of SSEA3^+^ cells from breast cancer stem cell subpopulation of MCF-7 and MDA-MB-231 cell lines further enhanced their tumor initiating capacity^49^. In addition, clustering of SSEA-4 by the monoclonal antibody promotes invasion ability of the breast cancer cell line through activation of cSrc and FAK^50^. Apart from these studies, the functions of SSEA-3 and SSEA-4 remain an enigma. Previously, our studies demonstrated that Globo H could promote angiogenesis through its interaction with Translin-Associated Factor X (TRAX), with consequent release and activation of phospholipase C β1 from TRAX to trigger Ca^2^^+^ mobilization^51^. In addition, Globo H acted as an immune checkpoint to facilitate the escape of tumor cells from immune surveillance by suppressing the activation of B-cell and T-cell^52^. These findings provided strong rationales for Globo H-targeted immunotherapy of cancer. Indeed, clinical trials of Globo H vaccine have generated promising results^53^.

In the present study, we found that high expression of either FUT1 or B3GALT5 was a significant risk factor for HCC relapse and high expression of B3GALT5 correlated with OS, but neither alone was an independent predictor for HCC recurrence or OS. Notably, multivariable analyses showed that a combination of high expression of FUT1 and B3TALT5 is an independent risk factor for recurrence and OS, in addition to vascular invasion and tumor size greater than 5 cm. The latter two were consistent with reports in many studies^6^. We are currently investigating the expression of Globo H, SSEA-3 and SSEA-4 in more HCC samples, in relation to these three glycosyltransferases and their clinical relevance.

The risk factors and incidence of HCC vary greatly according to geographic regions and ethnicities. The patients in our study were Taiwanese with HCC which were mostly (96.3%) associated with viral hepatitis. Thus, it remains to be determined if our finding of high expression of combined FUT1 and B3GALT5 as an independent risk factor for postoperative recurrence and OS in patients with resectable HCC will apply to HCC patients from different ethnicities or different etiologies, such as alcoholic and nonalcoholic steatohepatitis.

In conclusion, our finding of combined high expression of FUT1 and B3TALT5 as an independent risk factor for postoperative recurrence and OS of HCC represents a simple and valuable addition to the existing clinicopathological parameters for the prognostication of HCC. Surgical resection is the main stay of HCC treatment. However, high incidence rate of recurrence and lack of simple and reliable biomarkers for predicting postoperative recurrence is still a challenge. If confirmed in large cohort study, our findings provide an important armamentarium to fill the void. Furthermore, we have shown here that Globo H, SSEA-3 and SSEA-4 were expressed in HCC tissues but not in normal liver tissue. Thus, these GSLs and glycosyltransferase could be potential therapeutic targets for HCC.

## Materials and Methods

### Clinical specimens

Complementary DNA (cDNA) or total RNA extracted from surgically resected tumors, along with relevant clinical and pathological data from 135 patients with American Joint committee on Cancer HCC stage I to IV were obtained from Linkou Chang Gung Memorial Hospital and Taiwan Liver Cancer Network. Snap-Frozen HCC tissues used for GSL extraction were obtained from Tissue Bank of Tri-Service General Hospital, Taipei, Taiwan. Normal liver tissues and sections were obtained from trauma patients from the Tissue Bank of Linkou Chang Gung Memorial Hospital, Taoyuan, Taiwan. Informed consent was obtained from all subjects before their tissues were deposited. All methods were carried out in accordance with relevant guidelines and regulations and approved by Institutional Review Board of Chang Gung Medical Foundation, Institutional Review Board of Tri-Service General Hospital, and Biobank Ethics Committee of National Health Research Institutes.

### Immunohistochemistry

The primary antibodies used included the following: mAb VK9 for Globo H, (hybridoma provided by Dr. Govindaswami Ragupathi, Memorial Sloan-Kettering Cancer Center, New York, NY), anti-SSEA-4 (MC-813-70, R&D Systems, Minneapolis, MN) and anti-SSEA-3 (MC-631, R&D Systems, Minneapolis, MN). IHC analysis was performed on formalin-fixed, paraffin-embedded tissue. Sections (3 μm) on coated slides were deparaffinized and rehydrated, then subjected to antigen retrieval by autoclave at 121 °C for 5 minutes in antigen Retrieval AR-10 solution (BioGenex, Fremont, CA) for SSEA-3 and Globo H or in Target Retrieval Solution (Dako, Glostrup, Denmark) for SSEA-4. Sections were treated with H_2_O_2_ and then incubated with primary antibodies at 4 °C overnight, followed by staining with Super Sensitive Polymer-HRP Detection System (BioGenex, Fremont, CA) and developed by DAB substrate (BioGenex, Fremont, CA). Slides were counter-stained with Harris’ hematoxylin and mounted. Sections were examined by pathologists and digital images were captured by Aperio Scope AT Turbo Slide Scanner (Leica Biosystems) at 40X magnification.

### Quantitative reverse-transcription real-time polymerase chain reaction

Total RNA (1 μg) was converted to cDNA using high capacity cDNA reverse transcription kit (Applied Biosystems, CA, USA) according to the manufacturer’s instructions. The expression levels of FUT1, FUT2, B3GALT5 and ST3GAL2 were determined by using TaqMan Gene Expression Master Mix on an Applied Biosystems 7500 fast real-time PCR system. Glyceraldehyde-3-phosphate dehydrogenase (GAPDH) and glucuronidase beta (GUSB) were used as endogenous controls. The TaqMan probes for the detection of FUT1 (Hs00355741_m1), FUT2 (Hs00382834_m1), B3GALT5 (Hs00707757_s1), ST3GAL2 (Hs00911835_m1), GAPDH (Hs99999905_m1) and GUSB (Hs99999908_m1) were purchased from Applied Biosystems. Ten nanograms of cDNA sample were used for the qRT-PCR reaction following the manufacturer’s instructions. The fluorescent signals were analyzed by 7500 software v2.06.

### Matrix-assisted laser desorption ionization-time of flight (MALD-TOF) analysis of permethylated GSLs

GSLs were extracted from HCC tissue as described^54^. Briefly, frozen tissue (approximately 25 mg) was homogenized and extracted with methanol and chloroform and bath-sonicated for 30 min. After centrifugation, the supernatant was collected, and the pellet was extracted three times with the same solvent. The supernatants containing neutral and acidic GSLs were pooled. Neutral and acidic GSLs were further separated by anion-exchange chromatography. The purified GSLs were subjected to permethylation according to NaOH/DMSO method^55^. MS analyses were carried out on an SCIEX 5800 MALDI-TOF/TOF mass spectrometer using 2,5-dihydroxybenzoic acid as matrix. MS spectra were acquired in positive-ion mode and accumulated in 4000 laser shots with random sampling to increase the spectral reproducibility.

### Statistical Analysis

Data are presented as mean ± SD, count and percentages. The prognostic performance of genes was calculated by the receiver operating characteristic curve and the area under the ROC curve. The Youden index (sensitivity + specificity −1) was used to determine the optimal cut-off value for high versus low gene expression level. Survival curves were plotted by Kaplan–Meier method, with the log-rank test applied for comparison. The Cox proportional-hazards regression model was employed to evaluate the independent prognostic factors. All tests were two-sided and *P*-values < 0.05 were considered statistically significant. The statistical analyses were performed with Prism 5.0 (GraphPad Software, La Jolla, CA, USA) and MedCalc 14.8 (MedCalc Software, Ostend, Belgium) software.

## Electronic supplementary material

Supplementary information
