# Supplementary material for: High expression FUT1 and B3GALT5 is an independent predictor of postoperative recurrence and survival in hepatocellular carcinoma
Source: Sci Rep. 2017 Sep 7;7:10750. doi: 10.1038/s41598-017-11136-w (PMC5589766; doi:10.1038/s41598-017-11136-w)
Supplement: Supplementary file 1 — Supplementary information [file 41598_2017_11136_MOESM1_ESM.pdf]

High expression FUT1 and B3GALT5 is an independent predictor of postoperative recurrence and survival in hepatocellular carcinoma

Huan-Hsien Kuo[1,2], Ruey-Jen Lin[1], Jung-Tung Hung[1], Chung-Bao Hsieh[3], Tsai-Hsien Hung[1], Fei-Yun Lo[1], Ming-Yi Ho[1], Chau-Ting Yeh[4], Yen-Lin Huang[5], John Yu[1] and Alice L. Yu[1,6] *

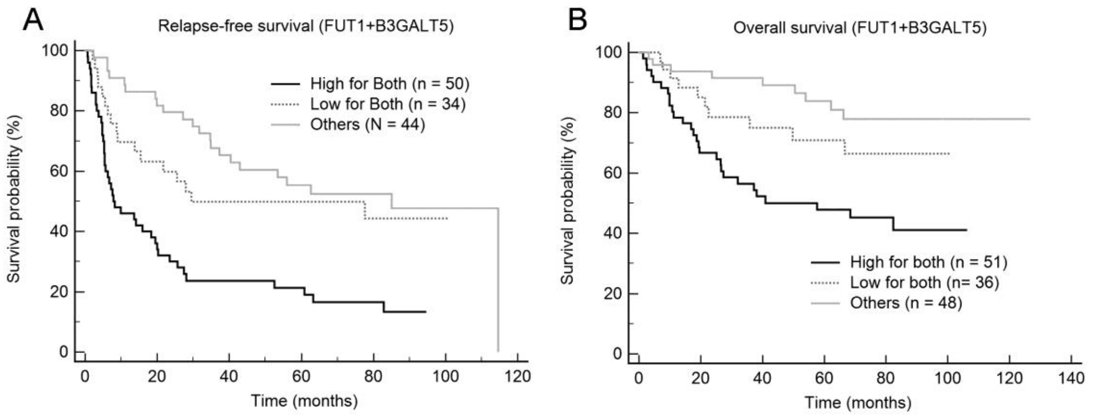

Supplementary Fig. 1s

Kaplan-Meier plots of relapse-free survival (A) and overall survival (B) for patients with HCC in relation to the risk groups according to the expression levels of FUT1 and B3GALT5.

Table S1: Association of FUT2 expression with clinical-pathological parameters in 135 patients with HCC

Variable		N	FUT2			
			Low	High	P value [a]	OR (95% CI)
Age	<55	56	20	36	0.197	1.0
	≧55	79	37	42		0.63 (0.31-1.27)
Gender:	Female	24	11	13	0.693	1.0
	Male	111	46	65		1.20 (0.49-2.90)
Virus infection :	None	5	1	4	0.375	1.0
	HBV	95	38	57		0.38 (0.04-3.49)
	HCV	29	14	15		0.27 (0.03-2.70)
	HBV+HCV	6	4	2		0.13 (0.01-2.00)
Liver cirrhosis:	No	76	32	44	0.975	1.0
	Yes	59	25	34		0.99 (0.50-1.97)
TNM stage :	I+II	102	42	60	0.665	1.0
	III+IV	33	15	18		0.84 (0.38-1.85)
Tumor size (cm):	≦5	88	40	48	0.298	1.0
	>5	47	17	30		1.47 (0.71-3.05)
AFP (ng/mL) [b]:	≦200	87	38	49	0.613	1.0
	> 200	46	18	28		1.21(0.58-2.50)
Tumor number [c]:	Solitary	84	33	51	0.324	1.0
	Multiple	50	24	26		0.70 (0.35-1.42)
Grade :	1-2	74	30	44	0.663	1.0
	3-4	61	27	34		0.86 (0.43-1.71)
Vascular invasion :	Absent	69	28	41	0.693	1.0
	Present	66	29	37		0.87 (0.40-1.73)
Metastasis :	No	122	49	73	0.138	1.0
	Yes	13	8	5		0.42 (0.13-1.36)
Relapse [d]:	No	48	23	25	0.439	1.0
	Yes	83	34	49		1.33(0.65-2.71)
Survival :	Alive	88	40	48	0.298	1.0
	Death	47	17	30		1.47 (0.71-3.05)

HBV hepatitis B virus, HCV hepatitis C virus, TNM tumor-node-metastasis, AFP alpha-fetoprotein, OR Odds Ratio. [a] Pearson Chi-square test. [b] data not available in 2 patients. [c] One patient with diffuse infiltrating tumor was excluded, [d] 4 patients with persistent tumor were excluded.
